# Improving the management and outcomes of preschool wheeze: protocol of a prospective multicentre cohort study

**DOI:** 10.1136/bmjresp-2025-003606

**Published:** 2026-02-05

**Authors:** Anne B Chang, Stephanie T Yerkovich, Steven McPhail, Hiran Selvadurai, Vikas Goyal, Shane George, Gabrielle B McCallum, Peter S Morris, Hannah O’farrell, Lesley Versteegh, Jonathan Grigg, Margaret McElrea, Sophie Worley, Terase Yerkovich, Leanne Elliot-Holmes, Joanna Williams, Keith Grimwood, Julie M Marchant

**Affiliations:** 1 Child and Maternal Health Division, Menzies School of Health Research, Darwin, Northern Territory, Australia; 2 Cough, Asthma and Airways Group, Australian Centre for Health Services Innovation and School of Medicine, Queensland University of Technology, Brisbane, Queensland, Australia; 3 Menzies School of Health Research, Darwin, Northern Territory, Australia; 4 School of Public Health and Social Work, Faculty of Health, Queensland University of Technology, 1. Australian Centre for Health Services Innovation and Centre for Healthcare Transformation, Brisbane, Queensland, Australia; 5 Metro South Health, Clinical Informatics Directorate, Woollongabba, Queensland, Australia; 6 Department of Respiratory Medicine, The Children's Hospital at Westmead, Sydney, New South Wales, Australia; 7 Discipline of Paediatrics and Child Health, The University of Sydney, Sydney, New South Wales, Australia; 8 Australian Centre for Health Services Innovation, Kelvin Grove, Queensland, Australia; 9 Queensland Children’s Hospital, South Brisbane, Queensland, Australia; 10 Emergency Department, Gold Coast University Hospital, Southport, Gold Coast, Australia; 11 School of Medicine and Dentistry, Griffith University, Brisbane, Queensland, Australia; 12 Child Health Division, Menzies School of Health Research, Darwin, Northern Territory, Australia; 13 Child Health Division, Darwin, Northern Territory, Australia; 14 Menzies School of Health Research, Casuarina, Northern Territory, Australia; 15 School of Medicine, Queensland University of Technology, Brisbane, Queensland, Australia; 16 Child and Maternal Health, Menzies School of Health Research, Casuarina, Northern Territory, Australia; 17 Academic Unit of Paediatrics, Queen Mary University London, London, UK; 18 Queensland University of Technology Centre for Children’s Health Research, Brisbane, Queensland, Australia; 19 Department of Respiratory and Sleep Medicine, Queensland Children’s Hospital, South Brisbane, Queensland, Australia; 20 School of Medicine, Queensland University of Technology Faculty of Health, Kelvin Grove, Queensland, Australia; 21 Parent Advisory Group Representative, Cough, Asthma and Airways Group, Australian Centre for Health Services Innovation and School of Clinical Medicine, Queensland University of Technology Faculty of Health, Brisbane, Queensland, Australia; 22 Asthma Foundation of the Northern Territory, Darwin, Northern Territory, Australia; 23 Parent Advisory Group Representative, Cough, Asthma and Airways Group, Australian Centre for Health Services Innovation and School of Clinical Medicine, Queensland University of Technology, Brisbane, Queensland, Australia; 24 School of Medicine and Dentistry, Griffith University, Griffith Health, Gold Coast, Queensland, Australia; 25 Child and Maternal Health, Menzies School of Health Research, Darwin, Northern Territory, Australia; 26 Australian Centre for Health Services Innovation and School of Clinical Medicine, Queensland University of Technology Faculty of Health, Brisbane, Queensland, Australia

**Keywords:** Paediatric asthma, Paediatric Lung Disaese

## Abstract

**Introduction:**

Preschool wheeze and asthma are associated with substantial morbidity and impaired future lung function. Yet, wheeze is unreliably reported with high disagreement (>50%) between parental and physician observations. Objectively defining wheeze and its reversibility could enable an earlier asthma diagnosis and improve preschool wheeze management.

Our primary aim is to determine in preschool children (aged 0.5–6 years) suspected of asthma whether adding WheezeScan to routine clinical assessment (vs assessment without WheezeScan) improves the diagnosis of asthma. Our primary hypothesis is that using WheezeScan in preschool children suspected of asthma is associated with increased definitive asthma diagnoses in this age group. Our secondary aims are to (a) examine the effect of using WheezeScan on patient-reported outcomes (PROs) and (b) healthcare costs. Our secondary hypothesis is that using WheezeScan in preschool children suspected of asthma is associated with improved quality of life without incurring additional healthcare costs.

**Methods and analysis:**

Our multicentre prospective cohort study involves recruiting 102 preschool children suspected of asthma. WheezeScan, a user-friendly digital device, incorporates artificial intelligence to objectively define wheeze and its response to bronchodilators. Over 6 weeks, parents/caregivers use the WheezeScan two times per day and whenever wheezing is suspected. If wheeze is detected, an inhaled short-acting β_2_-agonist is administered and WheezeScan determines if wheeze resolves thereafter.

Our primary endpoint is the proportion of children with a definitive asthma diagnosis, compared with baseline, based on the treating clinician’s assessment using WheezeScan data. Our secondary outcomes are PROs, reflecting generic health-related quality-of-life and cough-specific (if chronic cough present) outcomes and health costs.

**Ethics and dissemination:**

The Children’s Health Queensland Human Research Ethics Committee (HREC/23/QCHQ/100691) and the Queensland University of Technology Office of Research Ethics and Integrity approved the study. We will publish and share results with the academic and healthcare communities and relevant patient organisations.

**Trial registration number:**

Australian New Zealand Clinical Trials Registry ACTRN12623000904673.

**WHAT IS ALREADY KNOWN ON THIS TOPIC**

- Recurrent wheeze affects about 30–40% of preschool children, causing considerable morbidity, healthcare utilisation and costs, while also being associated with future lung function impairment. Approximately one-third of children with recurrent preschool wheeze are diagnosed with asthma later in childhood.

**WHAT THIS STUDY ADDS**

- This world first multicentre study seeks to address the need in preschool children to identify wheeze and reversible airway obstruction (RAO) accurately by using recently developed digital technology that objectively determines the presence or absence of wheeze. In so doing, it will also assess whether improving the diagnosis and management of RAO and asthma is associated with improved clinical and patient-reported outcomes without increasing healthcare costs.

**HOW THIS STUDY MIGHT AFFECT RESEARCH, PRACTICE OR POLICY**

- Identifying preschoolers with asthma from other causes of wheezing in this age group will fulfil a large unmet clinical need and research gap identified by our parents and Parent Advisory Group, as well as by an Australian and an international survey of clinicians and/or parents/caregivers.

## Introduction

 Wheeze is a continuous, usually high-pitched, breath sound accompanied by a prolonged expiratory phase in the respiratory cycle. It originates from air turbulence in the lower airways due to partially obstructed intrathoracic airways causing flow limitation.^1^ Important aetiologies in young children are acute lower respiratory infections (including bronchiolitis), asthma, an inhaled foreign body and other lower airway disorders. Up to 50% of children have had an episode of wheeze by the time they reach school age, with 30–40% experiencing further wheezing episodes.^2^ Irrespective of the underlying wheezing pathobiology, wheeze symptoms cause substantial morbidity^3^ and economic cost.^2^ In the USA, preschoolers with recurrent wheeze have approximately two times the rate of outpatient physician and emergency department visits for wheeze exacerbations and more than five times the hospitalisation rate of older children with known persistent asthma.^4^ Furthermore, preschool wheeze is also associated with future impaired lung function trajectories, even when the wheeze is transient.^5^

When children are old enough to perform spirometry, many with a history of recurrent wheeze as preschoolers demonstrate a clear pattern of reversible airway obstruction (RAO) after receiving an inhaled short-acting β_2_-agonist (SABA) and/or inhaled corticosteroids, thereby suggesting they have underlying asthma.^6 7^ A systematic review and meta-analysis^8^ that included 19 studies (n=1022 children) concluded ‘inhaled SABA treatment for acute wheeze/asthma symptoms showed beneficial effects in young children and infants’. The meta-analysis found treatment with inhaled SABAs in preschoolers with wheeze decreased respiratory and wheeze scores, decreased respiratory rate and increased oxygen saturation.^8^ Within 60 min of administering SABA, the standardised mean difference between groups (all favouring those on SABA) was: decreased respiratory score (−2.05 points, 95% CI −2.50 to –1.59), decreased respiratory rate (−0.86, 95% CI −1.30 to –0.41), increased oxygen saturation (0.56, 95% CI 0.16 to 0.95), decreased respiratory work (−0.80, 95% CI −1.60 to –0.00) and decreased wheezing score (−1.07, 95% CI −1.80 to –0.33). In the subgroup analyses of children aged <2 years, the systematic review found similar significant effects for all outcomes.^8^ Thus, it is unsurprising that preschool children with recurrent wheeze and symptomatic improvement following SABA treatment will be diagnosed with asthma and, in some cases, their history of wheeze may date back to infancy.^9^ Under these circumstances, asthma therapies are generally used, although there is substantial variability between guidelines,^3^ probably due in part to the lack of evidence and problems with objectively defining wheeze.

Nevertheless, current guidelines for diagnosing asthma recommend objective confirmation with spirometry or peak flow,^10^,^12^ although the exact criteria vary among guidelines. Effectively, the change in spirometry or peak flow represents objective documentation of RAO, the hallmark for diagnosing asthma. Lung function indices are rarely available for young children, and so in preschoolers, finding clinical evidence suggestive of RAO (ie, resolution of dyspnoea or wheeze with inhaled SABA)^13 14^ is often used as a surrogate measure. Nevertheless, this approach remains controversial as it requires accurate and objective detection of wheeze but often relies on parental detection and reporting.^3^

Despite the importance of detecting wheeze, there are high levels of disagreement (>50%) between parental and physician reporting of wheeze.^15^ This may cause underdiagnosis or overdiagnosis of asthma and lead to overuse or underuse of medications, children undergoing unnecessary procedures and high parental anxiety.^16 17^ Crucially, failing to recognise wheeze can delay diagnosis, especially if wheeze is recurrent, as it can defer appropriate treatment, which may lead to ongoing exacerbations, doctor visits, hospitalisations and an increased burden on parents/caregivers. In contrast, early recognition and treatment of asthma improves symptoms and quality of life (QoL).^10^,^12^

While detecting wheeze and its reversibility with SABA is not the sole predictor in the diagnostic journey for asthma in preschool children, it is still a crucial step. Addressing the poor reliability of detecting wheeze in young children^15 18^ is a clear clinical gap. Recently, this gap was highlighted by the European Respiratory Society (ERS) Taskforce statement on preschool wheezing disorders.^5^ Its importance is even greater in settings where reporting of respiratory symptoms is more challenging, such as in disadvantaged First Nations communities^19^ and in settings where there are diverse cultures and languages spoken.^18^

**Figure 1:**
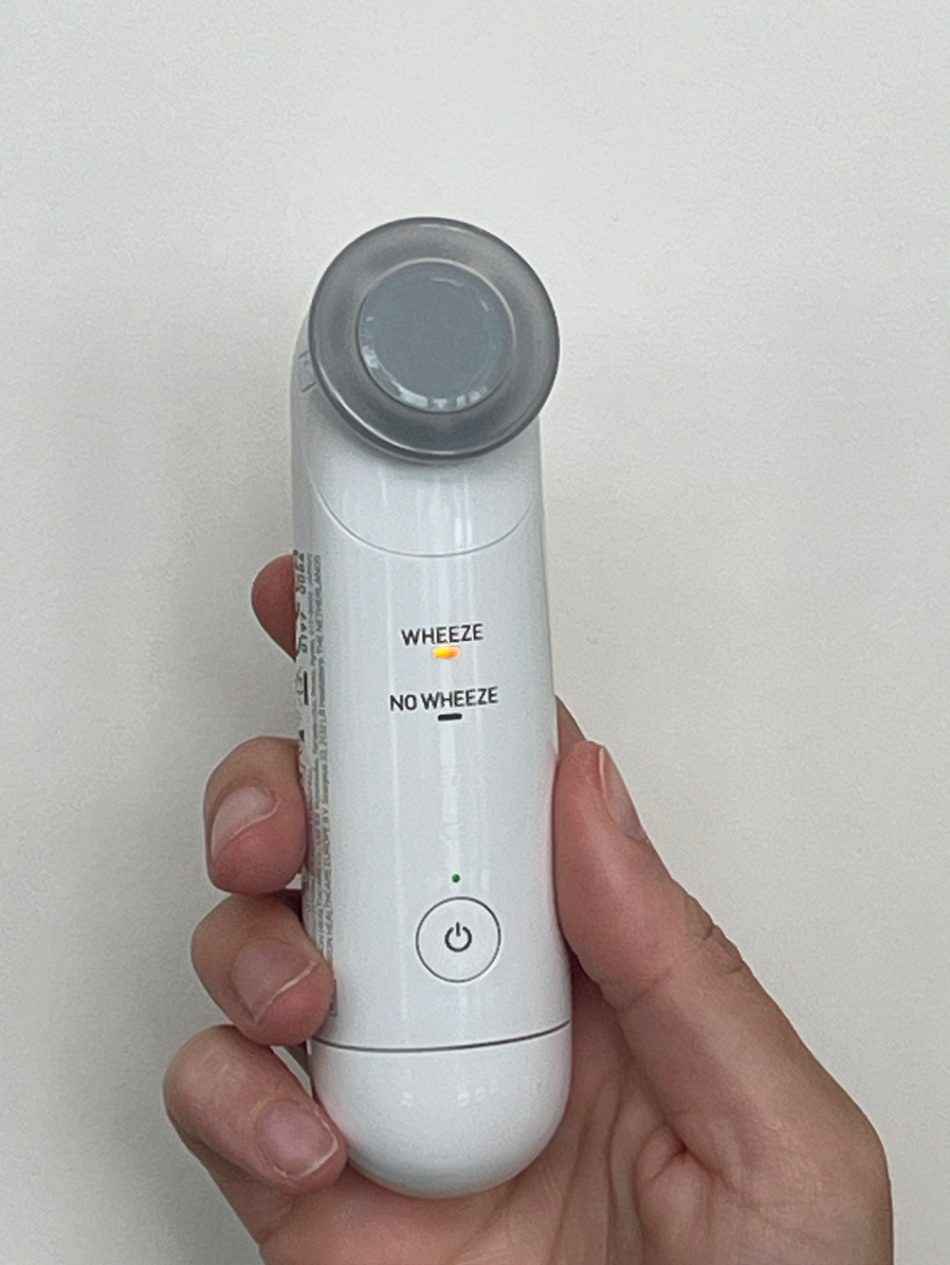
WheezeScan (Omron Healthcare, Japan). WheezeScan is the device we are using in our study. It is the world’s first clinically validated device^20^ that uses artificial intelligence to detect and differentiate wheeze from other breath sounds in young children aged 4 months to 7 years.

Reliable and objective detection of wheeze using digital technology in clinics during face-to-face visits and at home when applied by parents could lead to improved diagnosis from detecting wheeze that responds to asthma treatments. There are now clinically validated tools that can objectively define wheeze. One such tool is the WheezeScan (figure 1, Omron Healthcare, Japan), which is the world’s first clinically validated device^20^ that uses artificial intelligence to detect and differentiate wheeze from other breath sounds in young children aged 4 months to 7 years.^21^ For our study, we chose WheezeScan as its design is user-friendly, ergonomic, light and portable, allowing parents to have it with them at all times and to remove any uncertainty when breathing distress occurs. In a pilot study from Germany involving 20 children, parents detected wheeze in 22/708 (3.1%) routine two times per day recordings, but WheezeScan indicated wheeze was present in 140/708 (19.8%) of the recordings.^20^

In addition to detecting wheeze accurately to allow an earlier and more objective diagnosis of asthma in preschool children,^5^ having patient-reported outcomes (PROs) as trial endpoints is important for adoption of effective best practice, which is also relevant to patients and their families. PROs include parent’s perception of their own and their child’s QoL, and in the case of WheezeScan with its ease of use. Disadvantaged Indigenous children and those with cultural and language barriers have a higher burden of respiratory illnesses than other children in the community^22^ and, for equity, studies should make every effort to include these children, irrespective of obstacles that may arise.

Therefore, with so many gaps^5^ in the understanding and management of preschool wheeze, both clinicians and parents are left confused over whether or not wheeze is present in young children, if it indicates underlying asthma and, if so, how it should be treated.^16^ Our study addresses several of these key clinical and end-user identified gaps.

## Aims and hypotheses

Our primary aim is to determine in preschool children (aged 0.5–6 years) suspected of asthma whether adding WheezeScan to routine clinical assessment (vs assessment without WheezeScan) improves the diagnosis of asthma. Our primary hypothesis is that using WheezeScan in preschool children suspected of asthma is associated with increased definitive asthma diagnoses in this age group.

Our secondary aims are to examine the effect of using WheezeScan on: (a) PROs and (b) healthcare costs. Our secondary hypothesis is that using WheezeScan in preschool children suspected of asthma is associated with improved QoL without incurring additional healthcare costs.

Additionally, we aim to evaluate the perceived usefulness of WheezeScan for parents/caregivers using a qualitative evaluation.

## Methods and analysis

### Design

We are undertaking a prospective multicentre cohort study. Recruitment commenced from 1 February 2024 and is continiuing.

### Study settings and participants

#### Study sites

Our study sites are the: Queensland Children’s Hospital (Brisbane, Queensland and including Indigenous outreach clinics in rural and remote Queensland), Gold Coast University Hospital (Gold Coast, Queensland), Children’s Hospital at Westmead (Sydney, New South Wales) and Royal Darwin Hospital (Darwin, Northern Territory), all in Australia.

#### Inclusion criteria

Children (aged 0.5–6 years) undergoing review by paediatric specialists for suspected asthma (≥2 reported wheeze episodes, chronic (>4 weeks) dry cough or exertional breathlessness) and have ongoing symptoms.

#### Exclusion criteria

Exclusion criteria are: (a) current chronic wet cough, (b) previous specialist-diagnosed asthma, (c) previously enrolled, (d) unable to complete PROs or (e) participating in another trial with an active intervention that may affect or bias findings.

#### Recruitment

**Figure 2:**
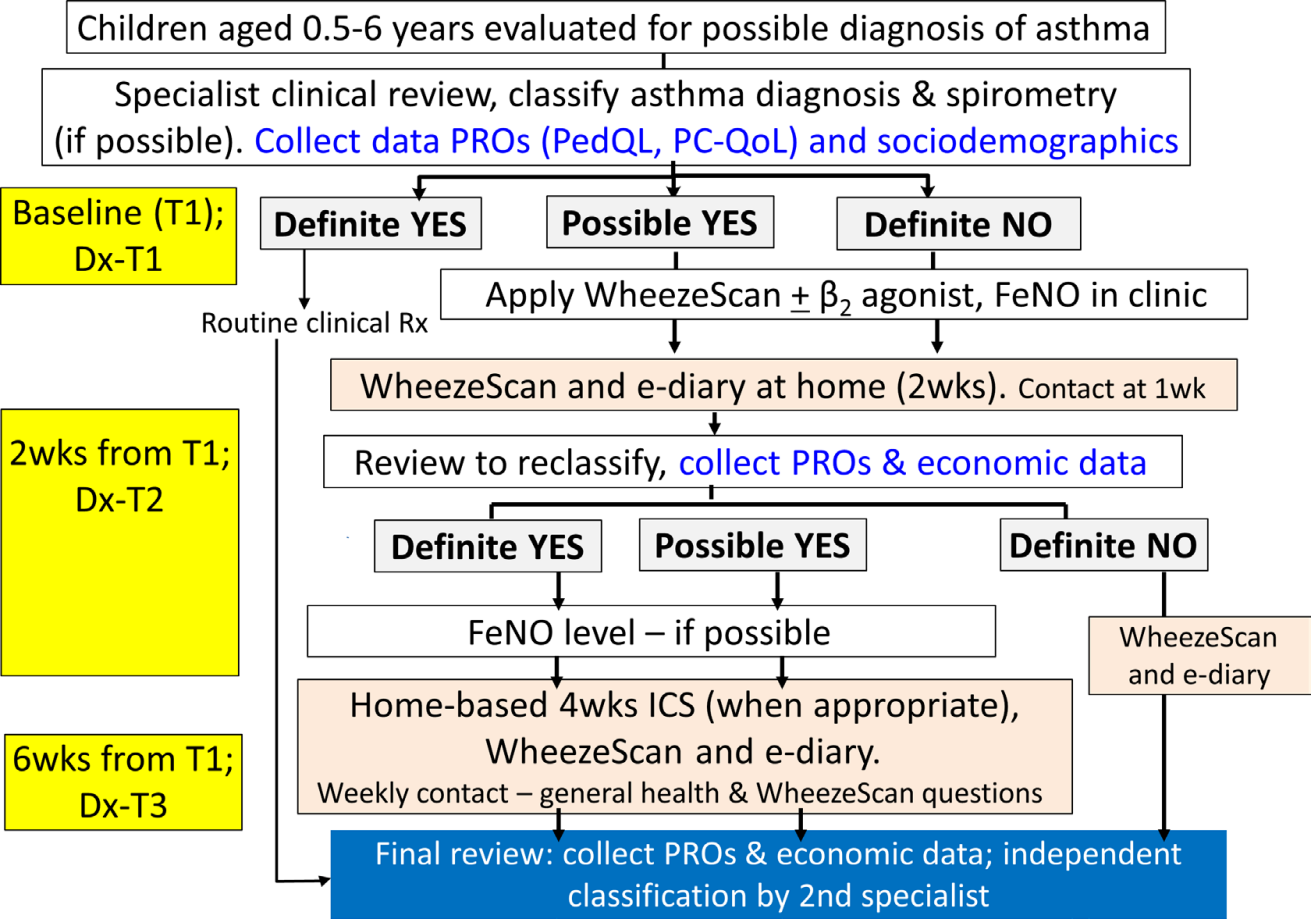
Study flow of our cohort study. Dx, diagnosis; FeNO, fractional exhaled nitric oxide; ICS, inhaled corticosteroids; PC-QoL, Parent Cough-Specific Quality-of-Life^29^; PedQL, Paediatric Quality-of-Life Inventory V.4^28^; PROs, patient reported outcomes; wks, weeks.

Eligible children are identified from general paediatric and respiratory clinics and the emergency department at each study site. At each site, those fulfilling the inclusion criteria are invited to participate in the study and informed consent is sought before enrolment. On obtaining informed consent, baseline data are collected, and a specialist clinical review is performed. The study flow is presented in figure 2 and the outline of procedures is summarised in table 1.

**Table 1:** Outline of study procedures

Assessment	Baseline (T1) At enrolment or prior to starting study	From baseline	
		2 weeks T2	6 weeks T3
	Window:	±4 days	±4 days
Screening for eligibility	✓		
Informed consent	✓		
Sociodemographic and medical history	✓		
Medical chart review	✓		
Clinical assessment	✓	✓ (optional)	✓ (optional)
Classify asthma (yes/possible/no)	✓ (primary specialist clinician)	✓ (primary specialist clinician)	✓ (primary and independent specialist clinicians)
PedsQL^28^	✓	✓	✓
PC-QoL^29^ (if chronic cough present)	✓	✓	✓
Health surveillance questions		✓	✓
Spirometry (if aged >4 years); pre-SABA and post-SABA where possible	✓	✓ (if seen)	✓ (if seen)
FeNO (if aged >4 years) once during study, if possible	✓	✓ (if seen)	✓ (if seen)
Analyse WheezeScan data		✓	✓
Parent WheezeScan utilisation question*			✓
NASSS-informed qualitative interviews		✓	or ✓
Collect results for full blood count, RAST, IgE (if available as part of clinical request)†	✓		or ✓
Contact parent—general health and WheezeScan questions about any problems encountered	Every week between T1 and T3		

* Parent utilisation question ‘The WheezeScan is useful in managing my child’s wheezing problem’ 5-point Likert (range from 1=strongly disagree to 5=strongly agree).

† Recorded for possible future studies.

FeNO, fractional exhaled nitric oxide; IgE, Immunoglobulin E; NASSS, non-adoption, abandonment, scale-up, spread and sustainability; PC-QoL, Parent Cough-Specific Quality-of-Life; PedsQL, Paediatric Quality-of-Life Inventory V.4; RAST, radioallergosorbent test; SABA, short-acting β2 agonist.

## Study protocol

### Data collection

Data are recorded by good clinical practice (GCP) trained researchers on case report forms as per our many trials.^23^,^27^

#### At baseline (timepoint 1 (T1))

We are collecting study PROs (Paediatric Quality-of-Life Inventory V.4 (PedsQL)^28^ (in all children) and Parent Cough-Specific Quality-of-Life (PC-QoL)^29^ (if chronic cough present) questionnaires, as done previously^23^ and sociodemographic and clinical data (eg, age, sex, family/household size, personal and family history of atopy, ethnicity (eg, First Nations), medications used, tobacco or e-cigarette exposure and immunisation history) from the parents/caregivers, medical notes and the Australian Immunisation Register.

As per routine practice, children are assessed clinically by a paediatric specialist, most of whom are respiratory specialists. Those who are classified as ‘definite yes’ receive routine clinical treatment and are not seen again until timepoint 3 (T3). All children have the WheezeScan applied and if wheeze is present, the child is given 400 µg (four puffs) of inhaled SABA (using a spacer±mask), the standard dose to define presence/absence of a bronchodilator response.^11^ After 10 min, the WheezeScan is used again to determine whether wheeze is still present and the results recorded. Whenever possible, fractional exhaled nitric oxide (FeNO) is measured, but the clinician is blinded to the result.

A member of the research team then teaches the parents/caregivers of those classified as ‘possible’ and ‘definite no’ how to use the WheezeScan and the e-diary, both of which are to be used routinely two times per day for the next 2 weeks and when wheeze is suspected by the parents/caregivers (eg, postphysical activity). The parents/caregivers are asked to give 400 µg of inhaled SABA (using a spacer±mask) if wheeze is detected and to then reapply WheezeScan 10 min later to determine and record whether RAO is present. If wheeze is still present, the parents are asked to give a further 400 µg of inhaled SABA (as above), and then to reapply WheezeScan again 10 min later, and record whether RAO is present.

#### Follow-up (timepoint 2 (T2))

The data from the children monitored by the WheezeScan are reviewed 2 weeks after the baseline visit. WheezeScan and e-diary data are analysed and used for reclassification of the ‘asthma’ diagnosis (Dx-T2). The ‘definite yes’ and ‘possible’ groups following the second reclassification step then commence, when considered clinically appropriate, 4 weeks of inhaled corticosteroid (fluticasone 100 µg two times per day, using a spacer±mask). The ‘definite no’ group continues in the study using the WheezeScan and administering 400 µg of inhaled SABA (using a spacer±mask) if wheeze is detected and then reapplying the WheezeScan 10 min later to determine and record whether RAO is present. As above, the SABA and WheezeScan steps may be repeated once. Also, at T2, PROs are collected.

#### Final visit (timepoint 3 (T3))

6 weeks post-T1, the children are then again reclassified using WheezeScan data and response to inhaled corticosteroids, the latter if used following T2 (Dx-T3). Also, at T3, PROs and economic data from all study participants are collected.

At the end of the study, a second independent respiratory specialist reviews clinical data, including available FeNO results and WheezeScan data, from all participating children and based on this information classifies the asthma diagnosis in the child (labelled as the independent classification [i-]). The independent person is blinded to the treating clinician’s assessment.

In addition, parents/caregivers of those who used the WheezeScan are also asked to give feedback on the usefulness of the device, using a Likert scale question. The question, framed with advice from our consumer’s group, is as follows: ‘The WheezeScan is useful in managing my child’s wheezing problems?’ The Likert scale ranges from 1 (strongly disagree) to 5 (strongly agree).

Study exit criteria are: consent withdrawn or intolerance of SABA or inhaled corticosteroids (as determined by study site doctors or treating clinicians).

### Outcomes and endpoints

- Asthma classifications
  - At T1, asthma classification is defined by the treating specialist clinician using clinical parameters.
  - For other timepoints (T2 and T3).
    ‘Definite yes’= presence of RAO defined by disappearance of wheeze using WheezeScan after 400 µg of inhaled SABA.
    ‘Definite no’= no wheeze documented.
    ‘Possible’=wheeze without clear response post-SABA or does not fit into the definite ‘yes’ or ‘no’ category.
  - Independent classification by a second specialist after reviewing the data at T3.
    ‘i-Asthma’= resolution of symptoms with treatment using WheezeScan data supported by FeNO >25ppb (if available).^11^
    ‘i-no’= no effect of treatment.
    ‘i-possible’= neither ‘yes’ nor ‘no’.
- Agreement between clinician and WheezeScan detection of wheeze at T1.
- PROs (PedsQL,^28^ PC-QoL^29^ (if chronic cough present)).
- Healthcare resource use and costs (healthcare system and family perspectives).
- Likert scale question relating to usefulness of WheezeScan at T3.
  See online supplemental file 1 for further description.

### Endpoints

#### Primary

Proportion of children with a definite diagnosis (yes/no) of asthma from T3 to T1, based on the classification provided by the treating specialist clinician.

#### Secondary

- Concordance of the asthma classification between the treating and independent specialist clinicians.
- Agreement between the clinical and WheezeScan detection of wheeze.
- Difference in PROs between the timepoints (T3−T1, T3−T2); subanalysed in accordance with whether inhaled corticosteroids were used.
- Composite measure of the ‘usefulness of WheezeScan’. We will consider WheezeScan clinically useful if: (a) WheezeScan assisted in a definite diagnosis (yes/no) or (b) PRO outcomes are superior in the WheezeScan-based versus independent asthma classification groups and (c) did not incur (net) healthcare costs during the study period.
- Healthcare resource use among the various classified groups (primary horizon at T3).

### Data monitoring, management and analyses

Database management is based in Brisbane, as with our previous/current multicentre studies,^23^,^2530^ and is also GCP adherent. Study data are stored and managed using REDCap electronic capture tools hosted at the Queensland University of Technology server, with an additional layer of security provided by multifactor authentication. Missing data will not be imputed. A detailed statistical analysis plan will be developed prior to the analyses. This includes adjusting for potential biases (eg, age), if necessary.

#### Analyses

For the primary aim we will compare the proportions using McNemar’s test.

For the secondary aims:

- Concordance of asthma classification (definite yes, definite no, possible) between the treating clinician and the independent specialist will be undertaken using weighted Kappa. Likewise, concordance between clinician and WheezeScan detected wheeze will be undertaken using Cohen’s Kappa.
- Change in PROs scores will be presented as means and SD and assessed by paired t-tests (assuming normal distribution) and the proportions with scores above the minimal clinically important difference (MCID) for each PRO in each group defined at T3 compared with T1.
- Composite measure of ‘usefulness of WheezeScan’ will be described and presented as numbers and percentages.
- Healthcare resource use associated with respiratory healthcare will be costed using actual costs (when known) or market rates from the perspectives of the healthcare system provider and families for each pathway (figure 2). Uncertainty estimates will be generated using bootstrap resampling (10 000 replications).

We anticipate reporting the last two secondary aims in a separate paper, as there will be a delay in the healthcare resource use analysis.

#### Sample size estimates

Our sample size is 102 children. This is based on conservative estimates of 15% being diagnosed with asthma at T1 and 30% at T3, with a correlation between the paired observations of 25%, which provides power>80% to detect a difference of 15% between the proportions. We will replace children with incomplete data and thus we expect to have complete data from 102 children. We conservatively estimate that 33% of children in our target group (preschool children with recurrent wheeze) have asthma (defined as RAO) and when adequately treated will be two times as likely to have significantly improved PRO above their MCID compared with when not treated due to wheeze misclassification known to be >50% in this age group.^15^

## Discussion

Our study fulfils a large unmet clinical need and research gap identified by our parents and Parent Advisory Group, as well as by Australian^31^ and international surveys of clinicians and/or parents/caregivers.^16^ Our study is based on using a novel digital tool, WheezeScan, which objectively detects wheeze in children. Currently, physicians determine if wheeze is present based on either their own observations when the child is seen in clinic, or on parental reports. Both approaches are problematic. First, these scenarios rely on understanding wheeze and then detecting its presence. The ability to detect wheeze on auscultation is dependent on physician expertise, with respiratory physicians being the most accurate, while the error rate for other specialist physicians is as much as 30%.^32^ Second, a clinical assessment takes only minutes, which may not reflect the child’s state in the prior days and weeks. Third, many groups have shown the disconnect between parental understanding and detection of wheeze compared with that of physicians, with studies revealing >50% of parental wheeze reports are inaccurate.^15^ Furthermore, parents frequently misidentified other respiratory noises (eg, rattles or upper airway sounds) as wheeze,^33^ and some parents stated they were unable to hear or detect wheeze in their children.^34^ Thus, the need for objective confirmation of the presence or absence of wheeze is obvious, especially in scenarios which influence clinical decision-making and treatment.

Our diagnostic approach (presence of asthma based on wheeze-defined RAO) is informed by a large international survey undertaken by the European Academy of Allergy & Clinical Immunology.^16^ The survey identified ‘*the most pressing problem with the diagnosis of wheezing in preschoolers is lack of diagnostic tools*’.^16^ It also found that response to treatment was the most favoured method for making a diagnosis for preschool wheeze.^16^

The lack of diagnostic tools provides a common clinical challenge faced by clinicians managing recurrent wheeze in preschool children. Our project’s primary aim is to address this problem by objectively showing wheeze reversibility with SABA administration. As spirometry cannot be reliably obtained in most preschoolers, detecting wheeze reversibility clinically is used to represent RAO, the hallmark of asthma and where inhaled corticosteroids improve clinical outcomes (hospitalisations, recurrent episodes and symptoms).^35^

### Potential study limitations

Potential limitations include the definition of RAO and its role in the classification of asthma. This is because defining RAO for the diagnosis of asthma in our target study population of preschoolers (aged 0.5–6 years) with recurrent wheeze is controversial and preschool wheeze is a heterogenous condition.^5^ Current ERS/American Thoracic Society (ATS) guideline criteria for defining bronchodilator responses (which indicates RAO) in adults rely on spirometry measured lung function indices where there is a >10% predicted increase in forced expiratory volume in one second (FEV_1_) or forced vital capacity, 15 min post administration of SABA.^10^ However, the ERS/ATS guideline does not specify the dose of SABA.^10^ Furthermore, guidelines in children differ; for example, the ERS clinical practice guideline for diagnosing asthma in children aged 5–16 years defines bronchodilator response as an increase in FEV_1_ of ≥12% and/or 200 mLs after a 400 µg dose of inhaled SABA.^11^ The 2024 Global Initiative for Asthma (GINA) guideline definition is a FEV_1_ increase of ≥12% post 200–400 µg of inhaled SABA (ie, volume change is not mentioned).^12^ Unfortunately, reliable spirometry is usually not possible in preschool-aged children and thus in our study, we are relying solely on objectively defined resolution of wheeze post-SABA to define RAO. Nevertheless, the definition of asthma used in our study’s target population (see inclusion and exclusion criteria) is consistent with the 2025 GINA guidelines as summarised by Venkatesan.^36^

Another limitation is our prospective cohort design rather than a randomised controlled trial (RCT). As such, our outcomes are at risk of bias. However, as far as we are aware, it is the first such study enabling a diagnosis of asthma based on objectively defined resolution of wheeze post-SABA and then for clinicians to initiate inhaled corticosteroids when clinically appropriate. Our design is therefore different to a recent RCT involving 167 children aged 4–48 months with a doctor diagnosis of wheezing in the preceding 12 months.^37^ This found no significant differences in their main outcomes (wheeze control and QoL) between the group who received the WheezeScan for at-home use for 120 days compared with ‘usual care’. However, the WheezeScan group had fewer unscheduled doctor visits (mean 1.6, SD 2.5) than the controls (mean 2.2, SD 3.3).^37^

Lastly, although the study duration was limited to 6 weeks, this timeframe should be sufficient to assess the primary outcome. Importantly, our study is not focused on children with established asthma. To determine the impact of using WheezeScan for asthma-specific outcomes requires a different study, which we are currently undertaking^38^ in parallel with this one.

### Significance statement

Recurrent wheeze in preschoolers is associated with substantial morbidity^3^ and economic cost.^2^ Furthermore, their morbidity is greater than in older children with persistent asthma, as they have two times the rate of outpatient physician and emergency department visits for wheeze exacerbations and more than five times the hospitalisation rate.^4^ Moreover, preschool wheeze is also associated with future impaired lung function trajectories, even when the wheeze is transient.^5^ In this protocol paper, we describe our study that addresses the clinical problem of wheeze being unreliably detected in preschoolers and consequently the possibility of preschool asthma being under-diagnosed. By employing a user-friendly ergonomic device employing artificial intelligence to objectively detect wheeze and its response to SABA, our study has the potential to improve asthma diagnosis (from detecting wheeze that responds to asthma treatments) and QoL in preschoolers with recurrent wheeze.

### Trial oversight

The study is monitored by the study sponsor, the Queensland University of Technology. We do not have an independent data safety and monitoring board.

### Patient and public involvement

Our study is codesigned with parents of children with preschool wheeze and our Parent Advisory Group in Brisbane. It was also discussed with, and supported by, the First Nations Reference Group (Darwin) and a representative from the Asthma Foundation of the Northern Territory, the region in Australia with the highest proportion of Indigenous Australians. As suggested by these consumer groups, we included PROs as an outcome measure. Since there is no validated questionnaire specific to preschool wheeze, we are using two validated PROs, a generic health-related QoL (PedsQL)^28^ and in children with a chronic cough, the PC-QoL,^29^ as research tools, which we have used previously.^23 39^ Two parents and a representative from the Asthma Foundation of the Northern Territory are invited to all our investigator meetings (held every 4–8 weeks) and ‘parents voice’ is a standing item in all these meetings.

## Dissemination

We will publish the results in a major medical journal and share the outcomes with the academic and medical community, funding and relevant patient organisations, including the Darwin First Nations Reference Group. Authorship eligibility guidelines will be used. We will not use professional writers. We currently do not have any plans to grant public access to the full protocol, participant-level dataset or statistical code.

## Supplementary material

10.1136/bmjresp-2025-003606 online supplemental file 1

## Data Availability

No data are available.
