# Supplementary material for: Improving the management and outcomes of preschool wheeze: protocol of a prospective multicentre cohort study
Source: BMJ Open Respir Res. 2026 Feb 5;13(1):e003606. doi: 10.1136/bmjresp-2025-003606 (PMC12878444; doi:10.1136/bmjresp-2025-003606)
Supplement: online supplemental file 1 [file bmjresp-13-1-s001.docx]

**Supplement**

Further description of methods, including patient-reported outcomes

- WheezeScan^TM^ (Omron Healthcare, Japan) is a device weighing just 60 grams. It has a diaphragm of micron-width material that detects low-volume wheezing and is combined with a high-definition quality microphone, a built-in noise cancelling system, and a specialised on-board computer. Its algorithm to detect wheeze is based upon artificial intelligence. The WheezeScan^TM^ device is simply held below the child’s clavicle for a maximum of 30-seconds until it signals “Wheeze” or “No Wheeze”.

In countries where WheezeScan^TM^ is available it pairs with its companion application, Omron Asthma Diary for mobile devices. The diary allows remote monitoring by healthcare professionals with details about the frequency of wheezing episodes sent from parents’ mobile phones. WheezeScan^TM^ is not yet available in Australia but is available in many European countries.^1^ In countries where WheezeScan^TM^ is marketed (e.g. in Europe), it stores the data in the device with measurement results and date/time stamps that are viewed in its Omron Diary. However, as the diary cannot be downloaded onto Australian phones, we are using a diary application linked with REDCap whereby parents record results in the diary at each use. Omron Healthcare (Japan), the manufacturers of WheezeScan^TM^ have loaned 150 WheezeScan^TM^ units without cost [https://www.omronbrandshop.com/WheezeScanTM/](https://www.omronbrandshop.com/wheezescan/).

- Fractional expired nitric oxide (FeNO): Whenever possible, FeNO measurements are undertaken using the handheld analyser (Niox Vero (Circassia, UK)), and are done at least twice within the same session to achieve results within 10% of each other as per the current guidelines, with the mean value recorded. FeNO is used to support the diagnosis of asthma although its validity and the cut-off thresholds are controversial.^2,3^ The European Respiratory Society clinical practice guidelines for asthma in children aged 5-12 years suggest FeNO >25 ppb as their cut-off^4^ and is the threshold we are using. However, the most recent British Thoracic Society guidelines recommend a cut-off >35 ppb to diagnose asthma in children aged 5-16 years with a history suggestive of asthma.^5^
- Patient-reported outcomes (PROs): Without a pre-school wheeze-specific quality-of-life (QoL) tool, we are using the Paediatric Quality-of-Life Inventory Version 4 (PedsQL) questionnaire^6^ for all subjects and the Parent Cough-Specific Quality-of-Life (PC-QoL) questionnaire^7^ for those with a chronic cough (as per our prior studies^8^). PedsQL^6^ is a
  23 item health-related generic validated multi-dimensional QoL tool designed for parental reports of their child’s QoL. The score is normalised giving a maximum score of 100.
  PC-QoL^7^ has eight questions providing summary scores of 1 to 7 with a minimally important clinical difference of 0.9.^7^ Higher scores indicate a better QoL.

**References**

1 WheezeScan. https://healthcare omron com/about-us/design-philosophy/wheezescan

2 Turner SW, Chang AB, Yang IA. Clinical utility of exhaled nitric oxide fraction in the management of asthma and COPD. Breathe (Sheff ) 2019; 15(4):306-316.

3 Collaro AJ, Chang AB, Marchant JM et al. Developing Fractional Exhaled Nitric Oxide Predicted and Upper Limit of Normal Values for a Disadvantaged Population. Chest 2023; 163(3):624-633.

4 Gaillard EA, Kuehni CE, Turner S et al. European Respiratory Society clinical practice guidelines for the diagnosis of asthma in children aged 5-16 years. Eur Respir J 2021; 58(5):2004173.

5 BTS/NICE/SIGN joint guideline on asthma: diagnosis, monitoring and chronic asthma management (November 2024) - summary of recommendations. Thorax 2025; 80(7):416-424.

6 Varni JW, Burwinkle TM, Seid M et al. The PedsQL 4.0 as a pediatric population health measure: feasibility, reliability, and validity. Ambul Pediatr 2003; 3(6):329-341.

7 Newcombe PA, Sheffield JK, Chang AB. Parent cough-specific quality of life: Development and validation of a short form. J Allergy Clin Immunol 2013; 131(4):1069-1074.

8 Goyal V, Grimwood K, Byrnes CA et al. Amoxicillin-clavulanate versus azithromycin for respiratory exacerbations in children with bronchiectasis (BEST-2): A multi-centre, double-blind, non-inferiority randomised controlled trial. Lancet 2018; 392(10154):1197-1206.
